# A Smart Technique to Remove Ruptured Inflatable Bone Tamp From the Vertebral Body in Balloon Kyphoplasty

**DOI:** 10.7759/cureus.15106

**Published:** 2021-05-18

**Authors:** Ioannis Papaioannou, Vasileios K Mousafeiris, Georgia Pantazidou, Thomas Repantis, Panagiotis Korovessis

**Affiliations:** 1 Orthopedics, General Hospital of Patras, Patras, GRC; 2 Otolaryngology - Head and Neck Surgery, General Hospital of Patras, Patras, GRC

**Keywords:** minimal invasive approach, kyphoplasty, spine fracture, inflatable tamp, rongeur forceps

## Abstract

Accidental rupture of the inflatable bone tamp is a rare but possible complication during balloon kyphoplasty. We describe an easy and minimal invasive technique to remove this foreign body from fractured vertebra. A 62-year-old female patient with severe osteoporosis had a low energy trauma and sustained burst fracture of the 12th^ ^thoracic (Th12) vertebra. The inflated bone tamp was not possible to be fully deflated and during the maneuvers to withdraw the balloon, it was disassembled and trapped under the distal end of working cannula, remaining within the bone cavity formed by balloon. Since no standard recommendation for this complication exists in current literature, we faced the dilemma of either leaving ruptured bone tamp in situ or removing it with a more extensive approach. We decided to use an alternative minimal invasive technique and managed to remove it through the right pedicle using a small size straight pituitary rongeur forceps under biplane continuous image intensifier and neuromonitoring. Subsequently, balloon kyphoplasty (BK) was performed through the left cannula accompanied with pedicle screw fixation of the adjacent vertebrae. The patient was followed up to our outpatient department for one year without complications. This extremely rare complication during BK consists of a challenge for spine surgeons and interventional radiologists. The described technique is relatively easy, safe, minimal invasive, time-saving and avoids further complications related with trapping of foreign bodies within the vertebral body.

## Introduction

Reported incidence of low-energy osteoporotic vertebral fractures in the United States is estimated to be more than 500,000 new cases per year, while an increase of more than 50% is expected by 2025 [1]. Nowadays, several percutaneous minimal invasive surgical (MIS) interventions such as vertebroplasty (VP) or balloon kyphoplasty (BK) are used operatively to treat vertebral body fractures with significant advantages compared to open techniques [2].

Percutaneous BK utilizes an inflatable balloon bone tamp to reduce the compressed upper vertebral endplate, restore the anterior vertebral body height and create a cavity in the fractured vertebral body for polymethacrylate (PMMA) injection. Extra-canal PMMA leakage is a common complication during BK with incidence rates of approximately 18.4% [3,4]. Leakage to the spinal canal occurs less often but it could be potentially disastrous with irreversible damage to spinal cord. BK has specific complications such as balloon rupture, retained balloon in vertebral body, partial needle retention, cortical fracture of vertebral endplates during balloon inflation, re-collapse following balloon deflation and transient hyperalgesia after PMMA injection [3-5]. Another extremely rare but potentially fatal complication is spinal infection (0.46%) associated with vertebral augmentation, which often necessitates major spine surgery [5]. Percutaneous short-segment “hybrid” MIS techniques consisting of the pedicle screw construct accompanied with BK in fractured vertebra, can be used to increase construction rigidity immediately in thoracolumbar spinal injuries [6].

We present a new, simple, minimal invasive and safe technique to remove broken balloon from fractured vertebra during BK. This technique allows the removal of foreign bodies from vertebral body with no additional risks and therefore the possibility of spinal infection is eliminated. This technical report describes in detail the removal of an entrapped inflated tamp during BK of 12th thoracic (Th12) vertebra fracture. 

## Technical report

**Figure 1:**
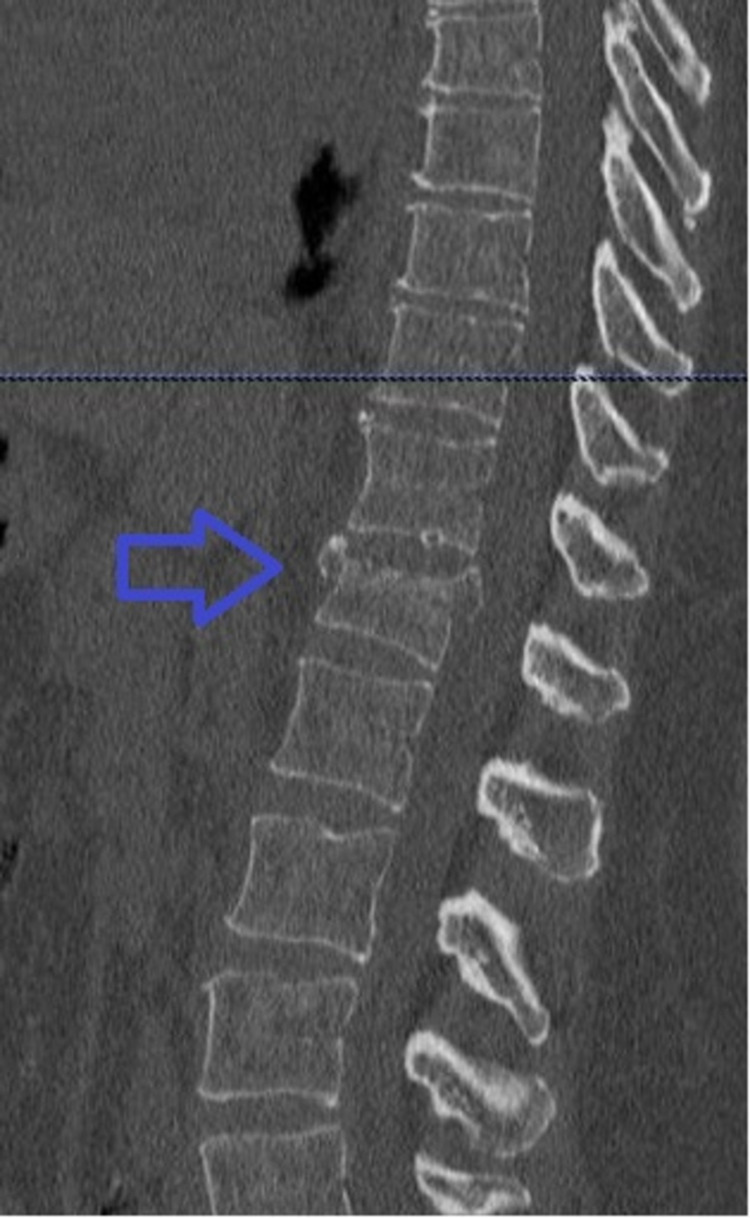
Saggital computed tomography reconstruction image. Blue arrow highlights the fracture of Th12 vertebrae.

**Figure 2:**
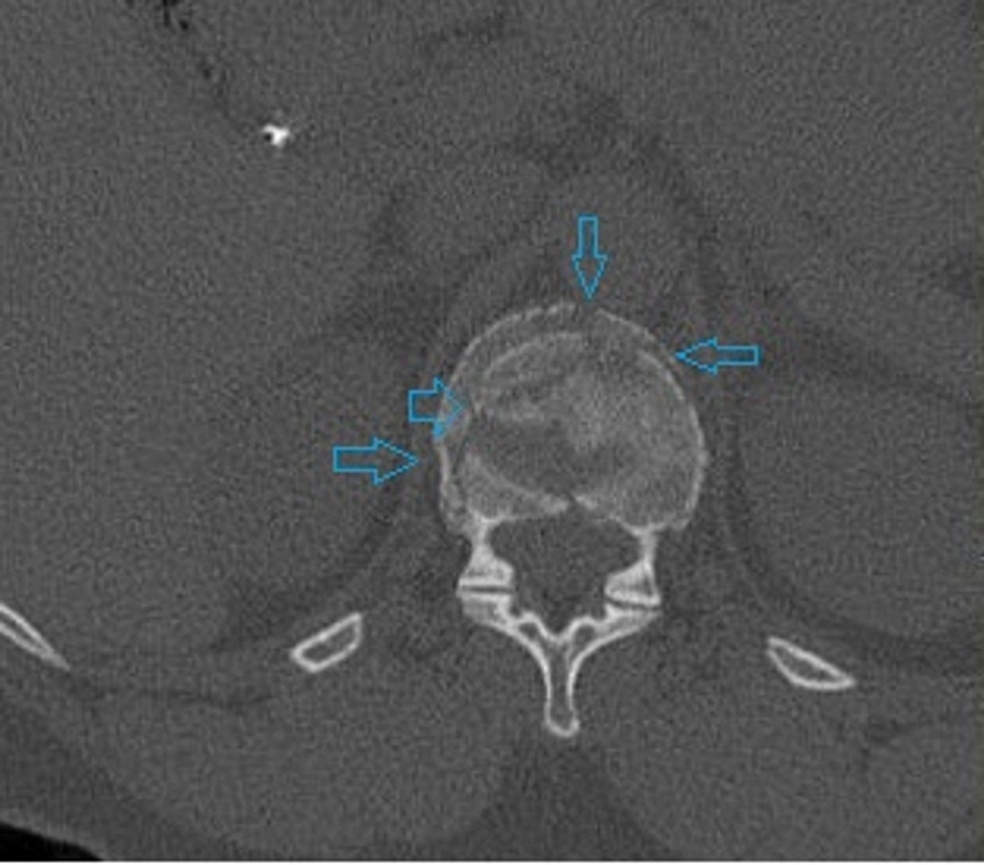
Axial computed tomography image. Small blue arrows demonstrate the comminution of the fractured vertebral body.

A 62-year-old female patient with severe osteoporosis was admitted to our emergency department after low energy injury. Physical examination showed tenderness to percussion at thoracolumbar junction without evidence of neurological impairment in the lower extremities. Computed tomography (CT) scan revealed burst fracture of Th12 vertebrae (Figures 1, 2).

**Figure 3:**
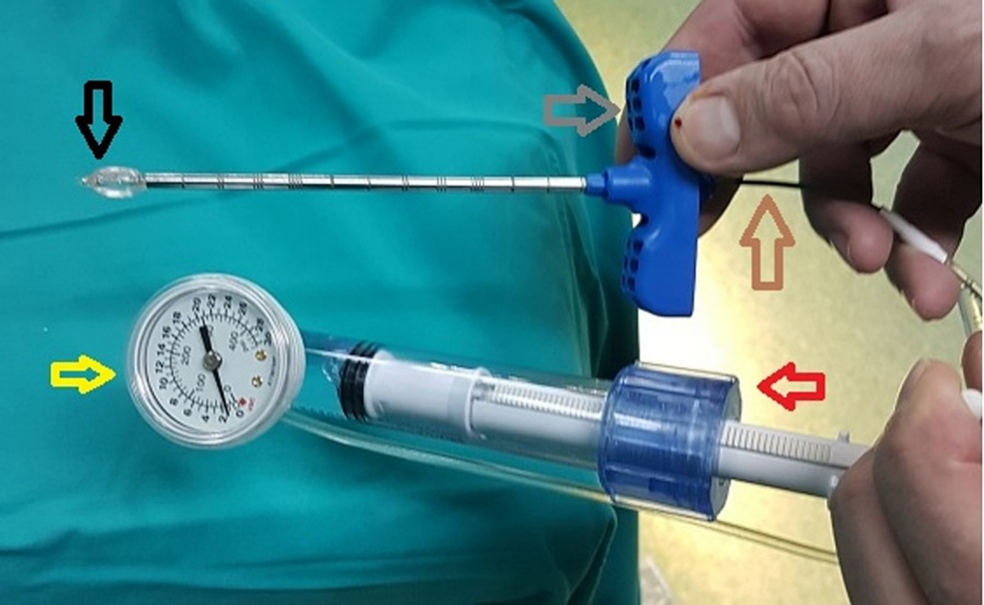
Photograph of the balloon kyphoplasty device used in this case. Red arrow shows the syrinx, yellow arrow demonstrates the myelographic liquid pressure index, brown arrow shows the flexible kyphoplasty tube, black arrow indicates the inflatable bone tamp, while grey arrow highlights the working cannula.

We decided to treat the patient with percutaneous pedicle screw fixation from the 10th^ ^thoracic to the first lumbar vertebrae (Th10-L1) accompanied with bilateral transpedicular BK of Th12 based on the location and the comminution of the fracture as well as the severe osteoporosis of the patient. Under general anesthesia, the patient was placed in prone position. A pair of 6.5 mm pedicle screws were successfully inserted in the pedicles of Th10, Th11 and L1 vertebrae under direct fluoroscopic guidance and continuous neuromonitoring. Subsequently, two access trocars with cannula were inserted in the pedicles of Th12 vertebra. Trocars were removed and a straight guidewire was inserted through the trocar cannula. Afterwards, a working cannula with a diameter of 5 mm was placed through the straight guidewire in each of the pedicles of Th12 vertebra. A hand drill was gently inserted to make access for the inflatable bone tamp. The inflatable bone tamp of this specific device has approximately 20 mm length and is connected with a flexible kyphoplasty tube and to the syrinx, which supplies the tamp with myelographic contrast liquid under appropriate pressure (Figure 3).

**Figure 4:**
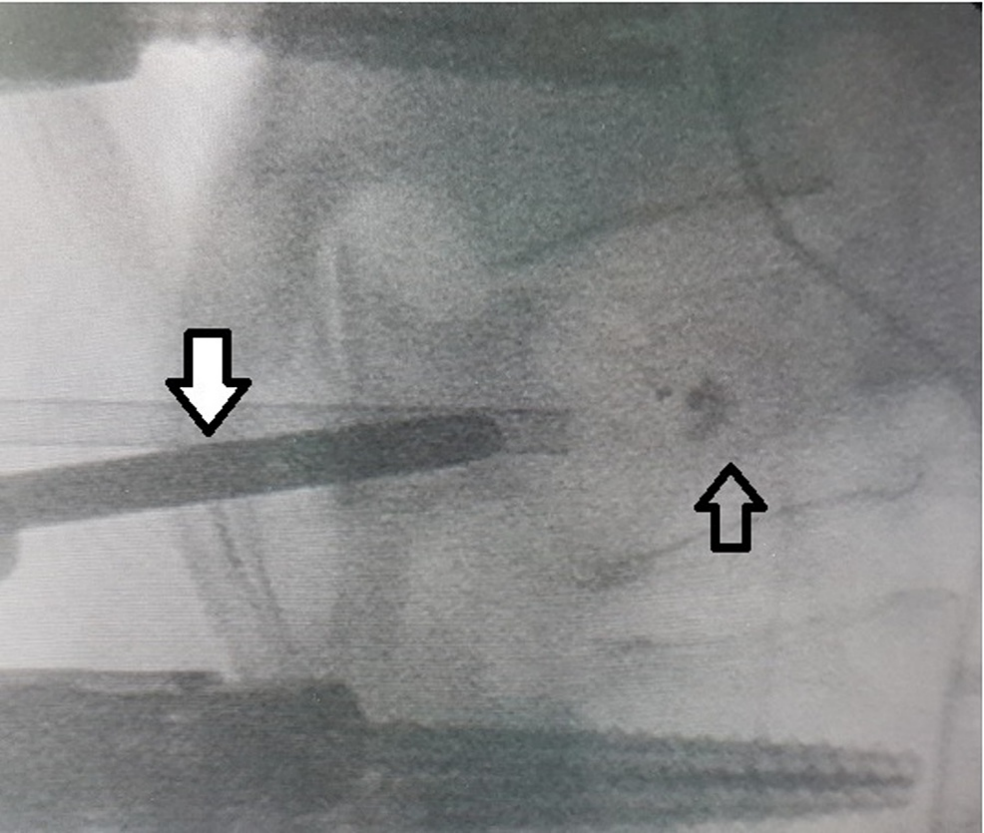
Intraoperative lateral image intensifier view showing the pituitary forceps entering the right pedicle (white/black arrow). The simple black arrow indicates the deflated bone tamp in the middle of the vertebral body with contrast liquid remnants.

In our case, the tamp was inflated up to 250 pounds per square inch (PSI) according to manufacturer's instructions, firstly to the right side and subsequently to the left side, creating thus a sufficient intraosseous cavity. The left balloon was easily deflated and removed after adequate cavity formation inside the Th12 vertebral body, while the right balloon was not possible to be deflated and became lodged at the distal tip of the working cannula. During the maneuvers to deflate and withdraw the tamp, it broke and was disassembled from the flexible kyphoplasty guiding tube, remaining within the void created by the balloon (Figure 4).

**Figure 5:**
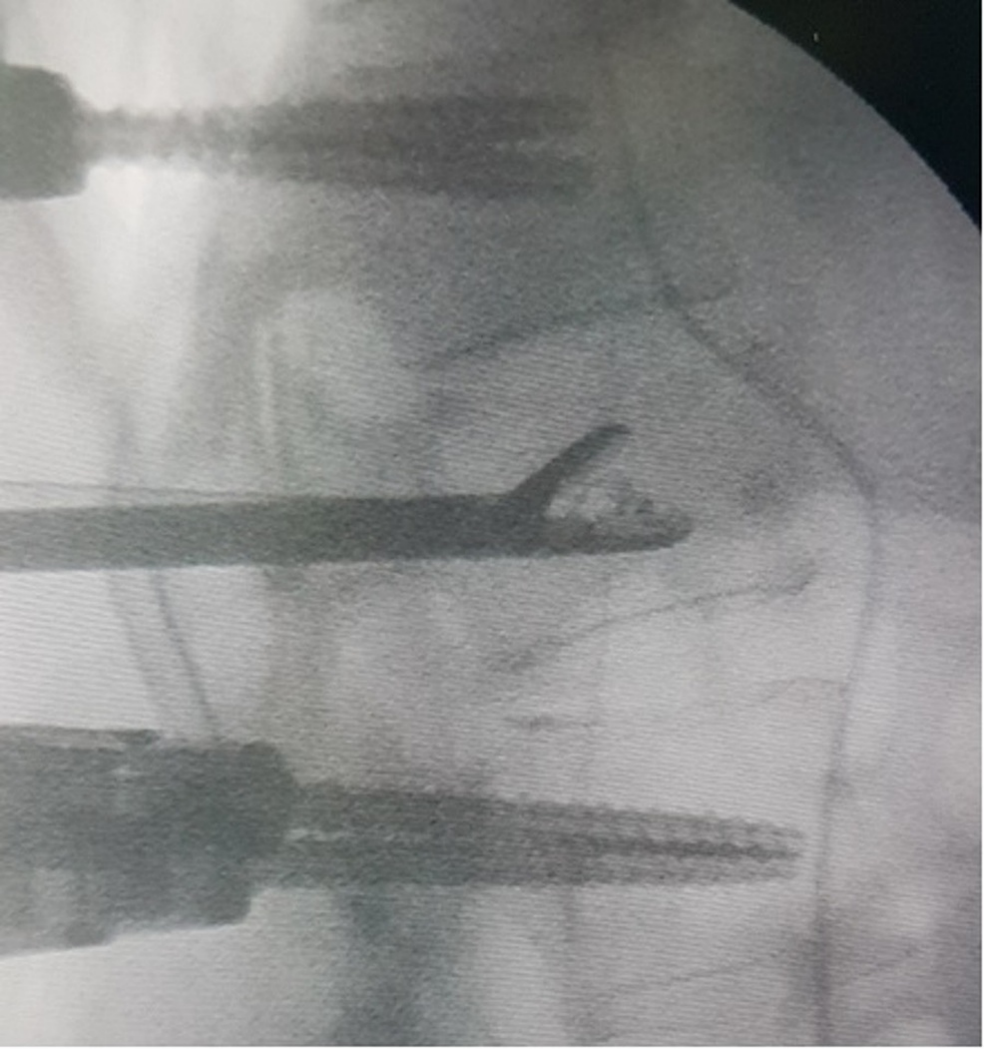
Intraoperative lateral image intensifier view showing the open forceps having grasped the bone tamp immediately before removal.

**Figure 6:**
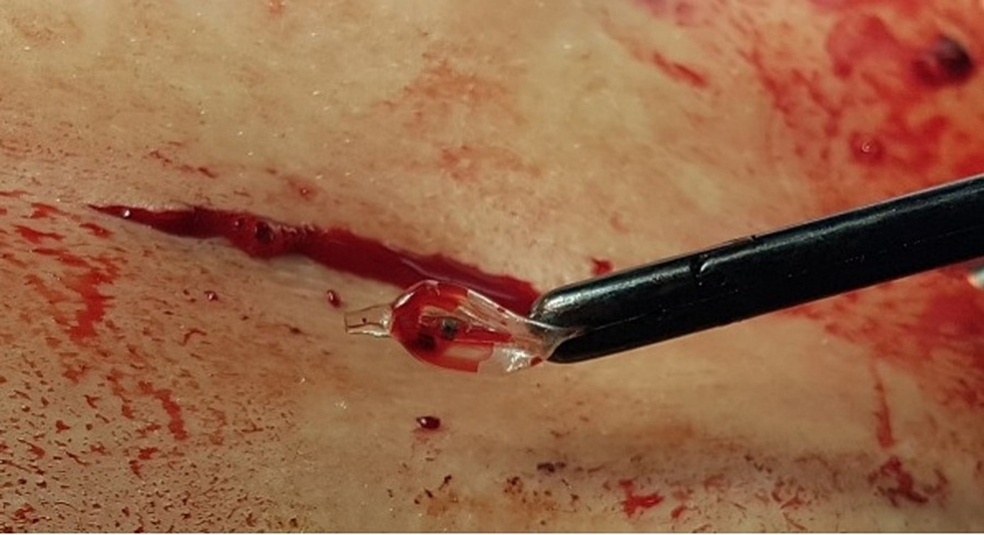
Pituitary forceps with the removed bone tamp. Note the small skin incision.

Since no standard recommendation exists to front this complication, the real alternatives were either leaving in situ the tamp as retained foreign object within the vertebral body [3,7] or removing it via an anterior [5] or posterior approach. Finally, the pedicle was dilated via the hand drill and using a small size pituitary rongeur forceps (10 millimetres total diameter) under biplane continuous image intensifier and neuromonitoring, we caught the deflated tamp and removed it (Figures 5, 6).

**Figure 7:**
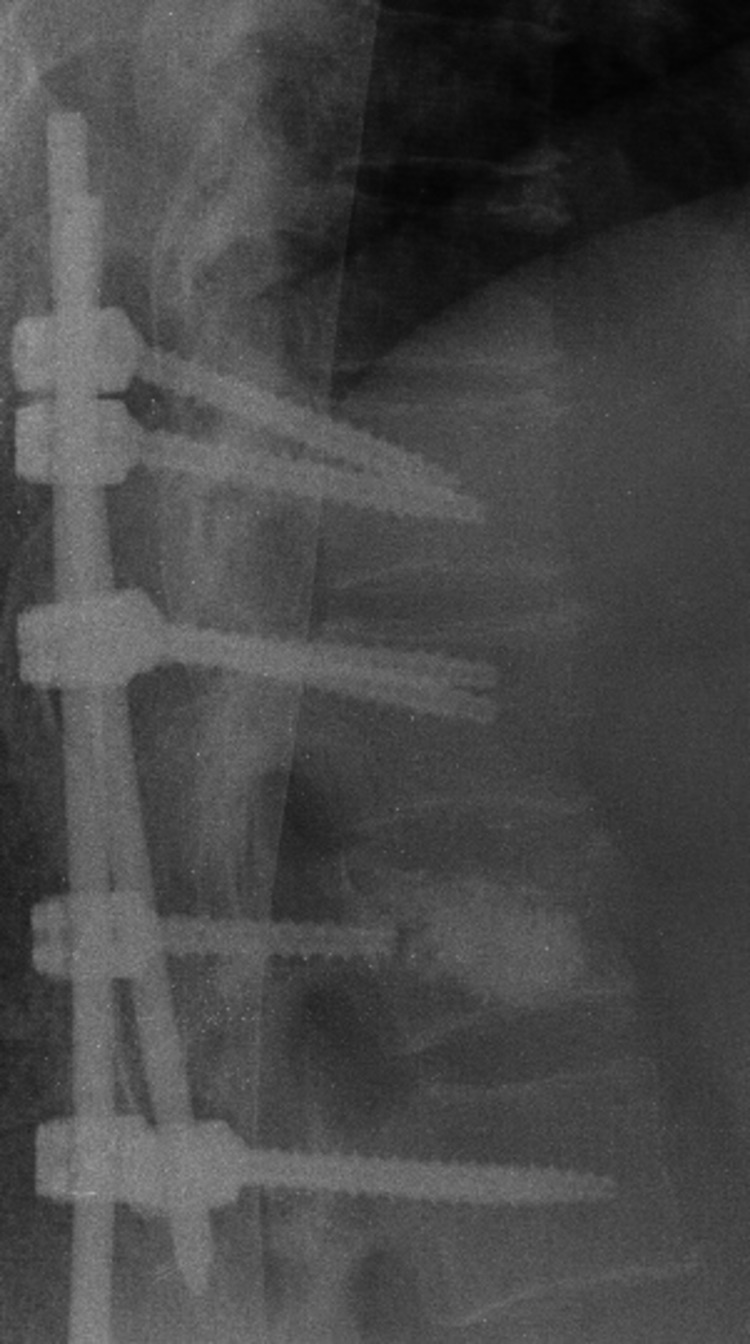
Lateral roentgenogram after balloon kyphoplasty and spinal instrumentation.

Subsequently, left-side BK was made without complications and the instrumentation was completed with connection of longitudinal rods and screws (Figure 7).

The patient was followed up in the outpatient clinic for 12 months without any neurological complications or infection.

## Discussion

The objective of MIS techniques in spinal surgery is to improve patient’s quality of life, reduce complications, blood loss and infection rate, aiming at a reinforced vertebral body accompanied with proper spine alignment. The most common complications associated with VP and BK are the following: PMMA leakage, peripheral & lung embolism, persistent pain, adjacent and remote fractures [6]. Established short-term complications of BK are also cortical fracture of vertebral endplates during balloon inflation, re-collapse of the vertebral fracture after balloon deflation, balloon rupture during inflation and transient hyperalgesia after PMMA injection due to the exothermic reaction of cement [3]. However, there are some rare and potential life-threatening complications associated with VP or BK, such as spinal infection that may pose serious dilemmas for spine surgeons [5]. Recently, Chalhoub et al described a new complication associated with BK lumbar fracture augmentation, where the bone tamp broke off inside the vertebral body and was impossible to be removed [3]. It is reported that the authors left balloon fragments in situ and completed the augmentation with PMMA [3], although the correctness of this choice in the specific study cannot be checked since no follow-up was mentioned and no conclusion can be settled. We describe the second case of this complication, although we also appose a “smart” and safe technique to remove the broken balloon tamp from^ ^vertebral body, under the same anesthesia via a posterior approach using one of the BK skin and pedicle portals. The already proposed risk factors for breakage and retention of balloon in the vertebral body are the following: increased pressure to inflate bone tamp, acute angle between balloon and working cannula, incomplete deflation of balloon prior to its removal, intrinsic physical properties of the balloon and laceration of it from sharp vertebral bone fragments that can rupture it even at low pressures [3,5,8]. In our case, we speculate that the causes for rupture of bone tamp were firstly the inability to sufficiently deflate the balloon probably due to construction failure and also surgeons’ maneuvers to remove the flexible tube accompanied with the connected bone tamp.

Shah et al. present a case of BK for osteoporotic lumbar fracture in an 82-year-old female patient, that was performed via a curved coaxial needle and balloon system [9]. In this case, the curved needle became lodged in the vertebral body and was impossible to be removed. The needle was subsequently cut off at the level of the pedicle and left as a retained foreign body. They followed the patient 12 months postoperatively, but no neurologic complications or complaints were reported. In contrast with Chalhoub et al [3] and Shah et al [9] who decided to retain bone tamp and needle respectively inside the lumbar vertebral body, we removed the completely broken and deflated bone tamp, because we were skeptic that this foreign object might act as source for a future spinal infection in this elderly woman. Spinal infection is a potential life-threatening situation, which requires a major surgery, prolonged hospitalization and increased health costs. Based on that data, it is important all spine surgeons to eliminate the risk factors for the development of this devastating complication. It is worth noting that Abdelrahman et al. [5] treated operatively 6 (0.46%) cases, which developed spinal infection following VP and BK, performing major surgery (debridement and corpectomy through anterior approaches combined with posterior instrumentation) [4], while they also reported a death before a planned revision surgery. Incidence of retained foreign objects (RFOs) after surgery has been estimated to occur at a rate of approximately one per 5500 surgical interventions [10-14]. Vast majority of reported RFOs are sponges and most of them have been removed atraumatically after being identified on routine postoperative imaging. Few foreign objects have not been removed after discovery, specifically in cases where removal would pose a greater risk for the patient than allowing a foreign body to remain in place [10]. Retrieval of broken spinal hardware can be a particularly difficult process and there is no single best approach to reacquire this equipment [11]. There are reports in the literature of retrieving broken Jamshidi needle fragments [12] and a fractured pedicle cannulated probe [13,14]. To our knowledge, our case may be the first, which reports a really “smart” technique to remove safely BK hardware without the necessity for a major surgery.

## Conclusions

We strongly recommend spine surgeons and interventional radiologists performing vertebral augmentation, to remove retained foreign objects due to the risk of impending spinal infection, which although rare, may complicate MIS surgery, particularly in elderly and high-risk immunocompromised patients. Our proposed technique is safe, minimal and not time-consuming. Spine surgeons should be aware of this alternative to remove a broken bone tamp from vertebral body.
